# A Boolean Approach to Linear Prediction for Signaling Network Modeling

**DOI:** 10.1371/journal.pone.0012789

**Published:** 2010-09-16

**Authors:** Federica Eduati, Alberto Corradin, Barbara Di Camillo, Gianna Toffolo

**Affiliations:** Department of Information Engineering, University of Padova, Padova, Italy; Center for Genomic Regulation, Spain

## Abstract

The task of the DREAM4 (Dialogue for Reverse Engineering Assessments and Methods) “Predictive signaling network modeling” challenge was to develop a method that, from single-stimulus/inhibitor data, reconstructs a cause-effect network to be used to predict the protein activity level in multi-stimulus/inhibitor experimental conditions. The method presented in this paper, one of the best performing in this challenge, consists of 3 steps: 1. Boolean tables are inferred from single-stimulus/inhibitor data to classify whether a particular combination of stimulus and inhibitor is affecting the protein. 2. A cause-effect network is reconstructed starting from these tables. 3. Training data are linearly combined according to rules inferred from the reconstructed network. This method, although simple, permits one to achieve a good performance providing reasonable predictions based on a reconstructed network compatible with knowledge from the literature. It can be potentially used to predict how signaling pathways are affected by different ligands and how this response is altered by diseases.

## Introduction

There is an increasing agreement of the scientific community in attributing complex disease such as cancer, diabetes, heart disease and autoimmunity to defects in signaling trasduction pathways. For instance, in the case of cancer, it is generally acknowledged that genetic mutations are involved in the onset of the disease, but its manifestation is at the pathway functional signaling level [1], [2]. Thus, an important step towards a dynamic understanding of the functions and behaviors relevant to a particular system is modeling protein interactions, by integrating available knowledge on signaling pathways with novel high-throughput protein expression data. Development of new therapies would benefit from models and methods able to predict the alterations induced on protein expression levels by different therapeutical agents. Recently, some pioneering efforts were accomplished by Li et al. [3] who developed a computational framework for a functional input-output description of the Toll-like receptor signaling and the identification of potential targets for its modulation, and by Mitsos et al. [4] who proposed a computational approach based on the experimental protocol introduced in [5] and a methodology to create cell-specific Boolean models as presented in [6], to evaluate drug actions on signaling pathways.

Evaluation and comparison of the performance of algorithms for network inference and data prediction is still an open issue. The Predictive Signaling Network Modeling challenge of DREAM4 competition provides an important contribution to this topic, by addressing the problem of signaling network inference from single-stimulus/inhibitor data for prediction of multi-stimulus/inhibitor data. The challenge arises from the question of generating a model from a network and data as defined in [6]: to this purpose, the organizers provided the topology of a canonical signaling pathway, derived from the literature, and a training set they have published in [5] monitoring the activity of seven phosphoproteins (AKT, ERK12, Ikb, JNK12, p38, HSP27, MEK12) at three time points (0, 30 minutes and 3 hours) during twenty five different perturbations consisting of combinatorial treatment with zero or one cytokine (TNFa, IL1a, IGF1, TGFa) acting as a stimulus and zero or one inhibitor (MEKi, p38i, PI3Ki, IKKi). Participants were asked to a) update the network b) predict the seven phosphoprotein levels in response to twenty pair-wise combinations of stimuli (TGF, IL1a, IGF1, TGFa+IGF1) and inhibitors (p38i+MEKi, PI3Ki+MEKi, p38i+IKKi, PI3Ki+IKKi). The corresponding measured levels were available to participants only after the disclosure of the best performing teams and were used by the organizers to evaluate the quality of predictions. Network and data are a subset of those used in [5] and in [6], all measurements were performed using Luminex xMAP sandwich assay as described in [5] and were affected by measurement errors due to technical noise (SD = 300), and biological noise (CV = 8%) [7].

It was emphasized that the submitted network, specific for the HepG2 cell line, had to include only nodes representing measured or manipulated elements (i.e. stimuli, inhibited proteins and measured proteins) and edges underlying predictions, and that predictions had to be based on the reconstructed network. As anticipated, the challenge was evaluated on the basis of quality of predictions and sparsity of the network. Reliability of predictions was quantified, for each protein p, by the Normalized Squared Error NSE(p):
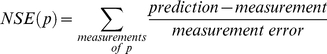
(1)NSE(p) was compared with a null distribution in which predictions were sampled at random from the measured values of each protein, p-values obtained for each protein were then combined in a Prediction Score: a larger score indicates greater statistical significance of the prediction. Finally, the Overall Score, which also considers the parsimony of the submitted network, was used for team ranking:
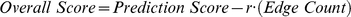
(2)where r is a parameter determined empirically by the organizers of the challenge as the minimum, over all teams, of the Prediction Score divided by the Edge Count.

In this paper, a simple data-driven method is presented, that was applied to this DREAM4 challenge. Network topology was reconstructed by inferring Boolean tables from training data, to establish cause-effect relationships characterizing the pathway in terms of links among ligands, inhibitors and proteins. Expression levels of the output proteins during multi-stimulus/inhibitor perturbations were then predicted by a linear combination of training data, in accordance with the reconstructed network.

## Methods

**Figure 1:**
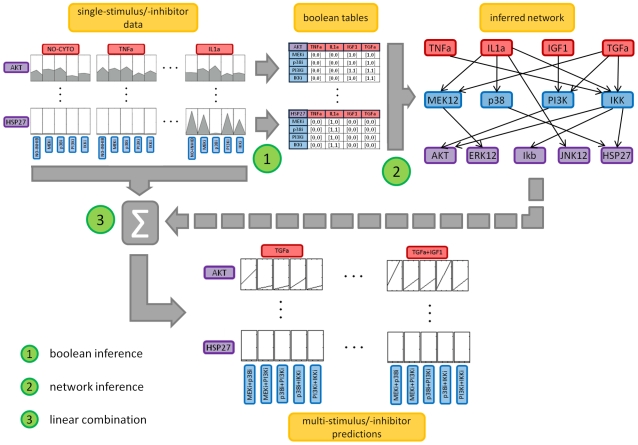
Workflow representing the 3 steps of the method. (1) Boolean inference of tables from single-stimulus/inhibitor experimental data, (2) network inference from the tables and (3) linear combination of single-stimulus/inhibitor data to predict protein activity level in multi-stimulus/inhibitor conditions, based on network structure.

The method consists of three steps (Figure 1) based on: 1) inference of Boolean tables from data to classify whether a particular combination of stimulus and inhibitor is affecting the protein, 2) reconstruction of a cause-effect network from Boolean tables, 3) prediction of test data by linear combination of training data, using rules based on the reconstructed network. The three steps are detailed in the following paragraphs by denoting, for a generic protein p (p = 1,…,7):

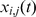
: protein level at time t collected after perturbation with stimulus i (i = 0,…,4 where i = 0 represents the condition without any stimulus) and inhibitor j (j = 0,…,4 where j = 0 represents the condition without any inhibitor);

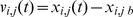
 : protein level with respect to the basal level (indicated by the suffix b);

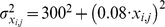
 : variance of the measurement error (provided by the organizers) associated to 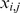;

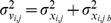
 : variance of the measurement error associated to 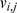.

### 1. Inference of Boolean tables

A table is built for each protein, having a column for each stimulus and a row for each inhibitor and containing in each cell a two-value vector 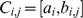 indicating how a particular stimulus/inhibitor combination affects the protein. 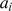 denotes the action of the stimulus *i* and 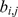 the action of stimulus *i*/inhibitor *j*, each quantized in two levels: 1 if action is significant, 0 if not.

Significant increase in protein level in response to a stimulus and significant decrease in response to an inhibitor are tested following [8], based on the measurement error distribution.

**Figure 2:**
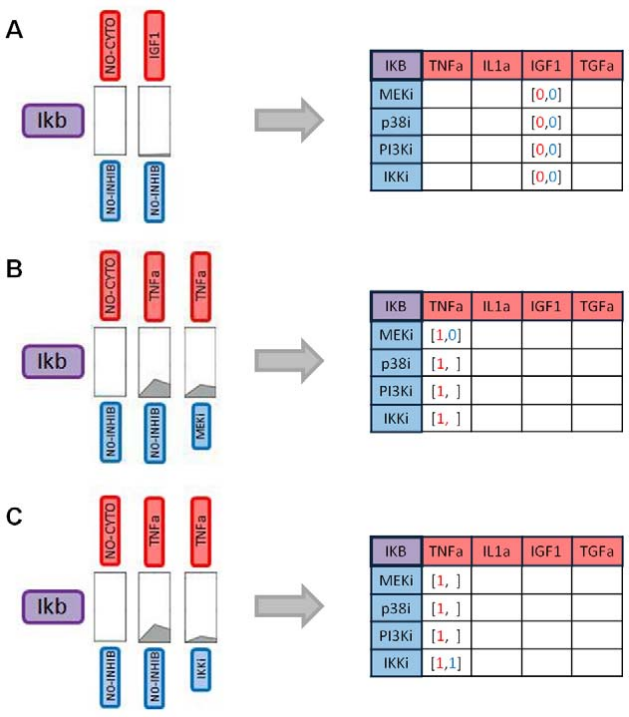
Boolean inference. Three examples are shown: A. stimulus IGF1 does not affect protein Ikb, B. stimulus TNFa affects protein Ikb but the presence of MEK inhibitor does not change the protein level, C. stimulus TNFa affects protein Ikb and the presence of IKK inhibitor decreases the protein level.

More precisely, for each stimulus, in absence of inhibitors, the increase of the protein activity (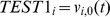) with respect to the reference, i.e. the condition with no stimulus and no inhibitor (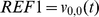), is considered significant if it exceeds k times the standard deviation of the measurement error, for at least one sample:
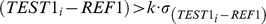
(3)where 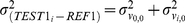 and k is a parameter to be set. As an example, Figure 2A reports the activity level of Ikb protein in the condition no stimulus/no inhibitor and in the condition stimulus IGF1/no inhibitor. The stimulus does not significantly affect the protein activity level, i.e. condition (3) is not satisfied, thus the first value of cells in the column corresponding to the stimulus IGF1 is set to 0, i.e. 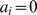. When condition (3) is not satisfied, as in Figure 2A, the effect of inhibitors is not considered and the second value of the cell (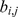) is set equal to 0 for all inhibitors j.

As a second example, Figure 2B shows the activity level of Ikb protein in the condition stimulus TNFa/no inhibitor. The stimulus affects the protein level, i.e. condition (3) is satisfied, thus the first value of cells in the column corresponding to stimulus TNFa is set to 1, i.e. 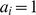. When condition (3) is satisfied, the effect of each inhibitor is analyzed. Denoting as reference the condition with the stimulus and no inhibitors (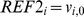) the action of each inhibitor (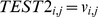) is considered significant if:
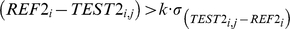
(4)where 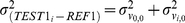. Figure 2B shows that if protein Ikb is stimulated with stimulus TNFa/inhibitor MEKi, condition (4) is not satisfied and the second value of the cell corresponding to stimulus TNFa and inhibitor MEKi is set to 0, i.e. 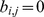. Whereas, with inhibitor IKKi (Figure 2C) condition (4) is satisfied, thus 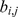 is set equal to 1. It is clear from the examples that the number of actions considered as significant is inversely related to the k value.

### 2. Network reconstruction

**Figure 3:**
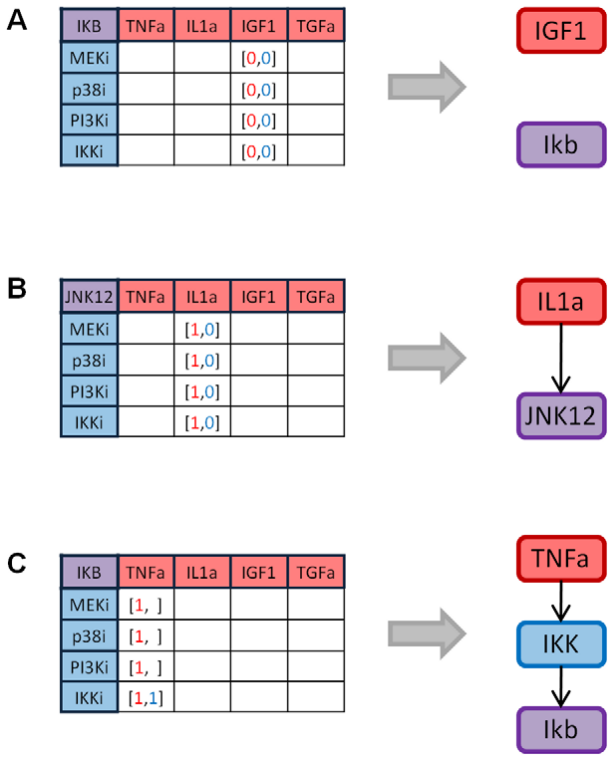
Network reconstruction. Three examples are shown: A. stimulus IGF1 does not affect protein Ikb, B. stimulus IL1a affects protein JNK12 but none of the inhibitors exerts a significant effect, C. stimulus TNFa affects protein Ikb and its action is mediated by protein IKK.

For each protein, a subnetwork is reconstructed from its Boolean table by adding:

- no links for stimulus/inhibitor combinations corresponding to [0,0] cells (example shown for protein Ikb under stimulation with stimulus IGF1 in Figure 3A);
- a direct link between a cytokine and a phosphoprotein if the column corresponding to that stimulus contained all [1,0] cells (e.g. IL1a→JNK12 in Figure 3B);
- a link passing through and inhibitor if, for that stimulus, there is a [1,1] cell in the row corresponding to that inhibitor (e.g. TNFa→IKK→Ikb in Figure 3C).

Subnetworks are then merged, and if, in the resulting network, a cytokine and a protein are connected both directly and indirectly, through an inhibitor, the direct link is pruned and not used for prediction. The Boolean tables are updated consistently.

### 3. Prediction

**Figure 4:**
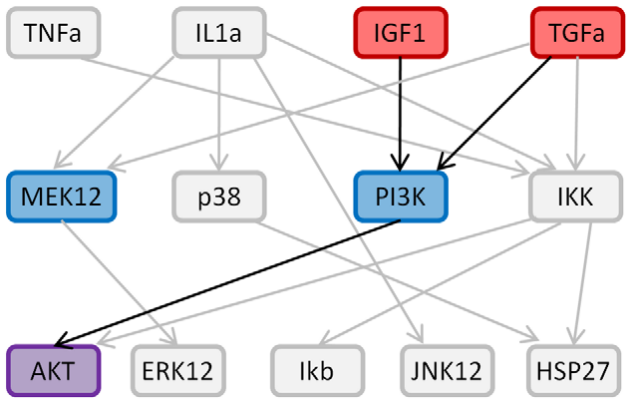
Subnetwork isolation for prediction. Example of the subnetwork considered when AKT value under stimulation with stimuli IGF1 and TGFa and inhibitors MEKi and PI3Ki had to be predicted.

To predict the phosphorylation level reached by a protein in combinatorial treatments with single or multiple stimuli/multiple inhibitors, the specific subnetwork is isolated. For example, to obtain the prediction of the activity of protein AKT in the condition with stimuli TGFa and IGF1 and inhibitors PI3Ki and MEK12i, the sub network composed by nodes TGFa, IGF1, PI3K, MEK12, AKT and links connecting them are isolated, as shown in Figure 4.

Depending on the subnetwork configuration, single-stimulus/inhibitor data are linearly combined according to the following formula:
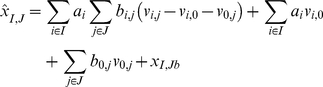
(5)where I and J denote the particular combinations of stimuli (e.g TGFa+IGF1) and inhibitors (e.g. MEKi+PI3Ki), respectively, for which prediction 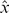 has to be made, 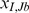 the basal level under this condition (given by the organizers) and 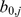 is assumed equal to 1 if 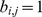 for at least one 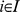. If none of 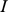 stimuli are active on the protein, i.e. 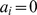 for all 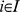, equation (5) reduces to:
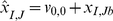
(6)


As an example, for the subnetwork shown in Figure 4, equation (5) predicts the activity of protein AKT with stimuli TGFa and IGF1 and inhibitors MEKi and PI3Ki as the sum of the activity level of protein AKT in the condition stimulus TGFa/inhibitor PI3K and in the condition stimulus IGF1/inhibitor PI3K. Since in this sum the effect of the inhibitor is considered twice, the activity level in the condition no stimulus/inhibitor PI3K is then subtracted. If, for a given protein, the reconstructed network predicts that some stimulus/inhibitor combinations do not affect its level, the reference conditions 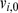 and 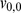 in equations (5) and (6) are evaluated by averaging the protein level measured in absence of stimulus/inhibitor with the protein level measured under these conditions.

### Implementation

The algorithm was implemented in Matlab. It requires as input arguments: single-stimulus/inhibitor data, the model of the measurement error, the value of parameter k and multi-stimuli/inhibitors combinations for which predictions are desired and provides as outputs: the reconstructed network with link ranking and predicted values.

## Results

### Network inference

**Figure 5:**
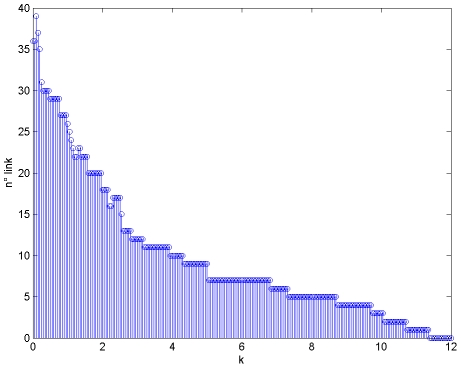
Influence of the parameter k on the number of links.

The choice of parameter k, used in the inference of Boolean tables to define the threshold of significance (equations (3) and (4)), obviously affects the number of links, as shown in Figure 5: with a high value of k only few links are included in the network, more are added if k decreases. In Table 1, selected links are ranked, according to the upper limit value of parameter k still allowing the presence of the link, from the most reliable (high value of k) to the less confident. A value of k equal to 2.5 was empirically chosen as threshold. It permitted to have an high number of true positives (i.e. links that are both in the canonical and reconstructed network) still limiting the number of false positives (i.e. links that appear in the reconstructed but not in the canonical network). Thus, the canonical network was used only to set a threshold valid for links to be selected, not as a priori information on which links are included in the network.

**Table 1:** Links in the network ranked according to the upper limit value of parameter k allowing the presence of the link.

LINK	k	Canonical network
‘IL1a→IKK→Ikb’	10.70	yes
‘IL1a→p38→HSP27’	9.74	yes
‘TGFa→MEK12’	8.71	yes
‘TGFa→PI3K→AKT’	5.01	yes
‘IGF1→PI3K→AKT’	4.38	yes
‘TNFa→IKK→Ikb’	4.34	yes
‘IL1a→JNK12’	4.31	yes
‘IL1a→MEK12’	3.91	no
‘IL1a→IKK→HSP27’	3.17	no
‘TGFa→MEK12→ERK12’	2.76	yes
‘IL1a→p38’	2.64	yes
‘TGFa→IKK→AKT’	2.55	no

The cause-effect network (after graph pruning), used for prediction, is shown in Figure 1. A direct connection between a cytokine (represented in red) and a measured protein (in purple), e.g. IL1a→Ikb, means that the cytokine stimulation significantly increased the activity level of the target protein. A connection through one of the inhibited proteins (in blue), e.g. TNFa→IKK→Ikb, means that the cytokine stimulates the target protein level, but if the halfway protein is inhibited the target protein level decreases with respect to the previous condition. All inferred links can be found in the canonical network but three: the one connecting IL1a and MEK12 also found by Saez-Rodriguez et al. [6] and the ones connecting IKK to AKT and HSP27. From Table 1, the connection between IKK and AKT is the last link is the ranking therefore it is the less confident. On the contrary the connection between IKK and HSP27 seems to be quite reliable.

### Prediction

**Figure 6:**
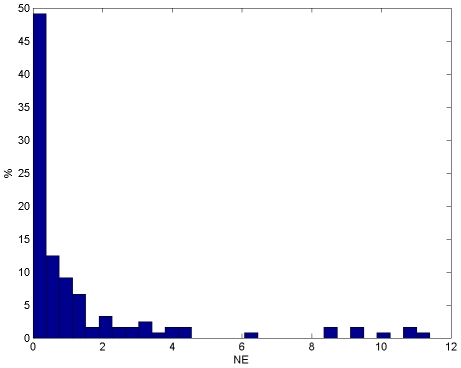
NE histogram. Mean and median NE values were 1.47 and 0.38 respectively.

The average Normalized Error (NE), i.e. the square root of NSE for each prediction, was 1.47 corresponding to an average deviation of prediction from measurement equal to 1.47 times the SD of the measurement error. In Figure 6, an histogram of single prediction NEs reveals that there were some outliers. Thus, the median NE was lower than the average NE and its value, equal to 0.38, indicates that the distance between the prediction and the real value was less than the 38% of the SD of the measurement error for the 50% of the predictions. Results for single proteins (Table 2) show that predictions are more precise for some proteins (e.g. p38 and HSP27), less precise for others, particularly for Akt, but in most cases the median is lower than the mean, indicating that outliers are distributed among proteins.

**Table 2:** Mean and median NE over all predicted values for each protein.

	TOT	AKT	ERK12	Ikb	JNK12	p38	HSP27	MEK12
mean NE	1.47	5.03	1.25	0.46	0.45	0.24	0.36	1.96
median NE	0.38	3.84	0.82	0.19	0.33	0.15	0.15	2.05

**Figure 7:**
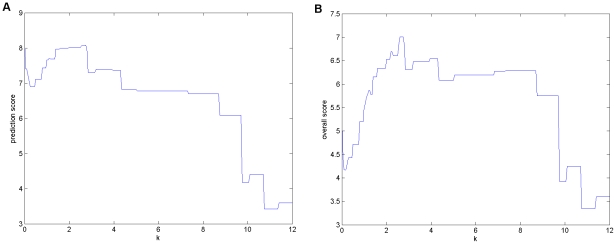
Influence of the parameter k on the performance of the method. (A) The Prediction Score is used to evaluate the statistical significance of the prediction, a greater value correspond to a better prediction. (B) The Overall Score consider also the parsimony of the network penalizing the Prediction Score with a cost per link. The method is robust to the value of k at least for 2<k<2.8.

In order to evaluate the role of parameter k on the performance, Prediction Score and Overall Score calculated from equation (2) by using r = 0.0827, which is the value evaluated by the organizers based on the results of all teams, were plotted for different values of k (Figure 7). Figure 7A shows that a high Prediction Score was obtained only for 1.4<k<2.7 indicating that a reliable network was necessary for the quality of predictions. In fact, low values of k, i.e. networks with many links, and high values of k, i.e. networks with few links, worsened the performance of the method in terms of Prediction Score. However, the Overall Score, which favored sparse networks, indicated a good performance even with high k values, as shown in Figure 7B.

## Discussion

In this paper, we present a simple method able to reconstruct, from single-stimulus/inhibitor protein data, cause-effect networks representing signaling pathways and to predict protein levels during multi-stimulus/inhibitor perturbations. This method, developed and applied to the Predictive Signaling Network Modeling challenge of DREAM4 competition, can be used to discover how signaling pathways are altered by diseases and to predict the effect of multiple agents/drugs. It uses a data driven approach, having Boolean (discrete logic) inference and linearity assumption as basic ingredients underlying network reconstruction and data prediction.

Boolean inference is appropriate to reconstruct the signaling network structured into input nodes (stimuli) intermediate nodes (inhibitors) and output nodes (phosphoproteins), particularly in situations like the one of the challenge, where the limited number of available samples and the lack of information on the stimulus format prevent the use of more sophisticated modeling approaches, e.g. based on differential equations and model identification. A cause-effect network connecting stimuli, inhibited and measured proteins, was reconstructed by a two step procedure: from single-stimulus/inhibitor data, a table was first built to code significant effects of stimuli and inhibitors on output proteins, which was then translated into links among nodes of the network according to very simple rules. Significance was defined with reference to the measurement error, by exploiting a method used in [8] to quantize time series expression data, e.g. a stimulus significantly affects an output protein if it is able to increase its level of a quantity that exceeds the uncertainty associated with the measurement of this quantity. The method needs information about the measurement error: in the case of the challenge a model relating the variance of the error to the expression level was provided by the organizers, in situations where this information is not available, it can be estimated from replicates [9]. A factor k, which multiplies the standard deviation of the errors, was introduced as a threshold to distinguish between not significant (to be explained in terms of measurement errors) and significant effects. The choice of k obviously affects network density, as shown in Figure 5: low k values favor dense networks and may lead to false positive links; whereas high k values cause sparse networks, potentially associated with false negative links. Thus, k was optimized using the available knowledge built in the canonical network. A value k = 2.5 was chosen, able to provide a network with most of the links reproducing direct or indirect connections also present in the canonical pathway. Therefore, a priori information built in the canonical network was only used to set parameter k. Anyhow, the described network reconstruction approach is strictly data-driven and thus usable even when no information is available: to set parameter k we are exploring different solutions, based on either the stability of predictions or the ranking of links. For example, active links can be selected following a method based on a compromise between false positives and false negatives based on a measurement error model, originally proposed to quantize gene expression data [8].

The reconstructed network was used to predict protein levels during multi-stimulus/inhibitor perturbations, by linear combination of single-stimulus/inhibitor data which, according to the network, exert significant effects on the proteins. Linearity assumption underlies the predictions, and this can be critical since interferences among different stimuli and/or different inhibitors are likely to occur in the real system. However, no information is available on whether and how interferences take place, therefore linearity is a sort of minimum working assumption, the role of which can be assessed a posteriori, based on the performance of the method in terms of reliability of predictions. Results indicate that the linearity assumption is reasonable, since the median of the deviation between true and predicted values is about 0.38 when normalized to the standard deviation of the measurement error. Performance is reasonably stable with respect to k values, reaching similar prediction scores for k in the range 2–2.8. Choosing a low value, resulting in a dense network, as well as a high value, resulting in a sparse network, deteriorates the quality of predictions. This supports the importance of using a realistic network to select the single-stimulus/inhibitor components to be linearly combined for data prediction.

In conclusion, the method we proposed provides a reliable solution to the problem proposed in the challenge. The method is simple, its implementation in Matlab has very low computational load but, despite of its simplicity, it is very promising and we are currently working on some refinements. As regards the definition of significance of the stimulus/inhibitor effect, we plan to introduce a criterion tailored for time series data, based on the area under the curve like in [10], instead of considering single data points. A second aspect regards the choice of parameter k, which is a critical issue of our method, in the situation where a priori knowledge of network density is not available/usable.
